# Notes of Cases

**Published:** 1895-06

**Authors:** 


### NOTES OF CASES.

### EQUINE HERMAPHRODITE.

BY EUGENE GOSS,
Junior Student K. C. V. C.

On April 28, 1895, I was called upon to operate upon a supposed male colt, but found instead a fine type of an hermaphrodite, four years of age, well bred, weight about one thousand pounds, in good health and flesh. The owner called me to amputate the penis, as it was unsightly when erect, protruding from the lips of the vulva about five inches, and was apparently perfectly formed.

The animal was extremely amorous and had been difficult to restrain, so I cast it as for castration, and examined the parts carefully. I found the mammæ well developed, with nipples of normal size for mare, and decided to operate as for a cryptorchid.

I commenced my incision at the base of the teat, on the posterior side, extending it about five inches, and making it large enough to admit my hand. After breaking down the fascia with my fingers and getting them into the inguinal canal, I found perfectly formed testicles just outside of the internal inguinal ring, the epididymis being largely developed.

After removing the testicles I concluded to await developments, and in about two weeks, as the animal had recovered, I decided to conclude the operation by removing the penis. After throwing and fastening, I inserted a small catheter into the urethra about seven inches, and, using my ecraseur, putting the chain around the penis an inch inside of the vulva, proceeded to take it off, leaving the catheter in the urethra and letting it protrude about an inch to prevent the urethra closing; packed the vagina with antiseptic gauze and left the animal to the care of the owner, who turned it out to pasture, where it made an excellent recovery without further care. There was very slight hemorrhage during the operation, and very slight subsequent swelling of the parts, and to-day the owner has an apparently perfectly formed mare.

---

### PROTRUSION OF THE JEJUNUM THROUGH THE VAGINA.

BY R. P. FEISER.

On April 15, 1895, I was called to attend a mare, the property of J. Wolf, near Abbottstown. On my arrival the mare was dead. Upon inquiry, the owner stated that in the morning he found the mare down and laboring, with about twelve feet of bowel protruding from the vagina. I held a postmortem and found the jejunum torn about five feet from the stomach, with the torn end protruding through a hole in the vagina near the symphysis pubis. The cervix uteri was dense and the walls of the vagina were also rigid. The os had not dilated. The fœtus was normal.

